# Directional Transport of a Bead Bound to Lamellipodial Surface Is Driven by Actin Polymerization

**DOI:** 10.1155/2017/7804251

**Published:** 2017-01-26

**Authors:** Daisuke Nobezawa, Sho-ichi Ikeda, Eitaro Wada, Takashi Nagano, Hidetake Miyata

**Affiliations:** Department of Physics, Tohoku University, Aramaki, Aoba-ku, Sendai, Miyagi 980-8578, Japan

## Abstract

The force driving the retrograde flow of actin cytoskeleton is important in the cellular activities involving cell movement (e.g., growth cone motility in axon guidance, wound healing, or cancer metastasis). However, relative importance of the forces generated by actin polymerization and myosin II in this process remains elusive. We have investigated the retrograde movement of the poly-d-lysine-coated bead attached with the optical trap to the edge of lamellipodium of Swiss 3T3 fibroblasts. The velocity of the attached bead drastically decreased by submicromolar concentration of cytochalasin D, latrunculin A, or jasplakinolide, indicating the involvement of actin turnover. On the other hand, the velocity decreased only slightly in the presence of 50 *μ*M (−)-blebbistatin and Y-27632. Comparative fluorescence microscopy of the distribution of actin filaments and that of myosin II revealed that the inhibition of actin turnover by cytochalasin D, latrunculin A, or jasplakinolide greatly diminished the actin filament network. On the other hand, inhibition of myosin II activity by (−)-blebbistatin or Y-27632 little affected the actin network but diminished stress fibers. Based on these results, we conclude that the actin polymerization/depolymerization plays the major role in the retrograde movement, while the myosin II activity is involved in the maintenance of the dynamic turnover of actin in lamellipodium.

## 1. Introduction

When the cell crawls on surfaces, it first protrudes the leading edge, and the protruded edge adheres to the surface. This is followed by a contraction of the cell body and subsequent dissociation of its rear [1]. The repetition of these steps makes the cell advance. The leading edge is a thin, veil-like membranous structure called lamellipodium that is filled with a dense, crisscrossed network of actin filaments (actin cytoskeleton). Between the lamellipodium and the nucleus is a region called lamellum. This region contains actin filaments and bipolar myosin mini-filaments; lamellipodium and lamellum are mechanically connected to each other by actin filaments [2, 3].

In lamellipodium, actin moves at a constant velocity toward the nucleus [4, 5]. This movement, called retrograde (actin) flow, continues to the end of lamellipodium, slowing down in lamellum. When a small object is bound to the surface near the edge of the cell, it is transported on the surface toward the nucleus [6]. The velocities of the retrograde movements of actin in the cell and the small object attached to the surface of the cell have been found to be the same [7–9]. Hence, it has been postulated that the object is coupled to the actin network through its interaction with transmembrane proteins. An example is the transport of a bead coated with glutamate receptor (mGluR5): the bead is initially executing a Brownian motion, but once engaged to the underlying cytoskeleton, it is transported toward nucleus [10]. Another example is a fibronectin receptor, integrin. Thus, when integrin beta-1 is bound by gold particles coated by fibronectin, it binds to the cytoskeleton and is transported rearward. The retrograde flow will exert a tensile force on the linkage between integrin and actin cytoskeleton strengthening the linkage [11]. This positive feedback must be important in the crawling of the cell.

The origin of the force driving the retrograde movement has been attributed to the polymerization of actin and/or the motor function of nonmuscle myosin II. In lamellipodium, the fast-growing (barbed) end of an actin filament is facing toward the front edge of lamellipodium [12–14]. Actin filaments growing toward the leading edge eventually hit and push the leading edge. Then, the filaments will receive a reaction force from the leading edge. If the actin cytoskeleton is firmly linked to the adhesion substrate through integrin, the lamellipodial membrane will protrude; if the link is disconnected or slips, the reaction force will push back the actin network, resulting in the retrograde flow. In neuronal growth cone the link (clutch) involves the immunoglobulin superfamily cell adhesion molecule [15] or the L1 cell adhesion molecule [16]. The latter molecule indirectly interacts with the actin cytoskeleton through a molecule, ankyrin_b_. The regulation of the linkage between the cytoskeleton and the adhesion substrate by the clutch mechanism is thought to be important in growth cone steering [17] or initiation of neuritogenesis [16].

In lamellum, myosin II forms bipolar filaments and those filaments together with actin filaments generate a contractile force in lamellum [18]. This force is transmitted to lamellipodium, because lamellum is mechanically coupled to lamellipodium by actin filaments, and the force will pull the actin filament network in the lamellipodium, causing the retrograde flow. The myosin-dependent retrograde flow has been experimentally shown [19]. When the actin filament network is linked to the adhesion substrate, the contraction of lamellum will pull the cell body thereby contributing to the forward translocation of the cell body. As described below, the actin-based or myosin II-based mechanisms seem to operate in the retrograde flow in cell-dependent fashion.

A study using a highly motile fish epidermal keratocyte has demonstrated that the cell speed is regulated by the linkage of cell adhesion molecules to the adhesion substrate as well as that of actin cytoskeleton to the clutch [20]. Thus, a firm linkage of the adhesion molecule to the adhesion substrate plus full engagement of the clutch will result in the fast protrusion and the fast movement of the cell. In the slow moving cell the receptor-adhesion linkage is weak and slips, resulting in the backward transport of the actin cytoskeleton. This will reduce the velocity of the protrusion of the leading edge, resulting in the slower advancing motion. In keratocyte, the sum of the rate of the protrusion and that of the retrograde flow is almost the same between the fast-moving and slow moving keratocytes, suggesting the regulation of the coupling between the polymerization rate and retrograde flow rate by the engagement of the clutch and the strength of cell adhesion. In a subtype of sea urchin coelomocyte, independent contribution of the actin polymerization and myosin II activity to the retrograde flow has been demonstrated [21]. When this cell was treated with an inhibitor of myosin II light chain kinase to reduce the activity of myosin II, the retrograde movement was halted in the cell interior but continued near the cell peripheral region. Inhibition of actin polymerization with cytochalasin D (CytD) partially slowed down the retrograde flow of actin cytoskeleton with the appearance of a cell fringe devoid of actin filaments. Based on these observations, the authors concluded that the two mechanisms, the pushing by actin polymerization at the front and the pulling by myosin-driven contraction of more central part of the cell, independently contribute to the retrograde flow.

In the present study, we measured the velocity of the polystyrene bead, which had been coated with poly-d-lysine and attached to the tip of lamellipodium of Swiss 3T3 fibroblast. Swiss 3T3 fibroblast has been used for several decades in the study of cell motility; its motility is between that of keratocyte and coelomocyte. With this system, we evaluated the relative contribution of the actin-dependent and myosin II-dependent mechanisms in the retrograde flow by using inhibitors specific to actin turnover and myosin II activity.

## 2. Materials and Methods

### 2.1. Chemicals

Dulbecco's modified Eagle medium (DMEM) was obtained from Nissui Pharmaceutical Co., Ltd. (Tokyo, Japan). DMEM without glucose, fetal bovine serum (FBS), penicillin-streptomycin, and l-glutamine were obtained from Thermo Fisher Scientific (Yokohama, Japan). Carboxylated polystyrene beads (1 *μ*m) were obtained from Polysciences, Inc. (Warrington, PA, USA). Poly-d-lysine, mouse monoclonal anti-myosin light chain antibody, formaldehyde, poly(oxyethylene) octylphenyl ether (Triton X-100), and jasplakinolide (Jasp) were obtained from Sigma-Aldrich Japan (Shinagawa, Tokyo, Japan). Plasmid of the red fluorescence protein (pDsRed monomer) fused to actin (hereafter called RFP-actin) was obtained from Clontech (Kusatsu, Shiga, Japan). The transfection reagent (Lipofectamine® 2000), rhodamine-conjugated phalloidin, and Alexa 488-labeled anti-mouse IgG were obtained from Invitrogen (Yokohama, Kanagawa, Japan). The (−)-blebbistatin, Y-27632, CytD, latrunculin A (LatA), and concanavalin A were obtained from Wako Chemical Co. (Osaka, Japan). A 2-[4-(2-hydroxyethyl)piperazin-1-yl]ethanesulfonic acid (HEPES) and biotin N-hydroxysulfosuccinimide ester (biotin-Sulfo-OSu) were obtained from Dojindo Laboratories (Kumamoto, Japan). Fibronectin fragment III (7–10) was obtained from Itou Life Science (Moriya City, Ibaraki, Japan). A 1 *μ*m, microsphere labeled with neutravidin (avidin-beads) was obtained from Molecular Probes (Eugene, OR, USA).

### 2.2. Cell and Cell Culture

Swiss3T3 fibroblast was obtained from JCRB Cell Bank (Ibaraki, Osaka, Japan). Cells were cultured in DMEM supplemented with 5% FBS, 1% penicillin-streptomycin, and 2 mM l-glutamine. Cells were placed in a CO_2_ incubator (NAPCO model 5410, Precision Scientific, Chicago, IL, USA) at 37°C, 5% CO_2_, and 90% humidity. Prior to the experiment, cells were subcultured overnight in the fresh cell culture medium; cells were plated in a custom-made chamber (Hirata chamber; see below) and the chamber was placed in the incubator.

**Figure 1:**
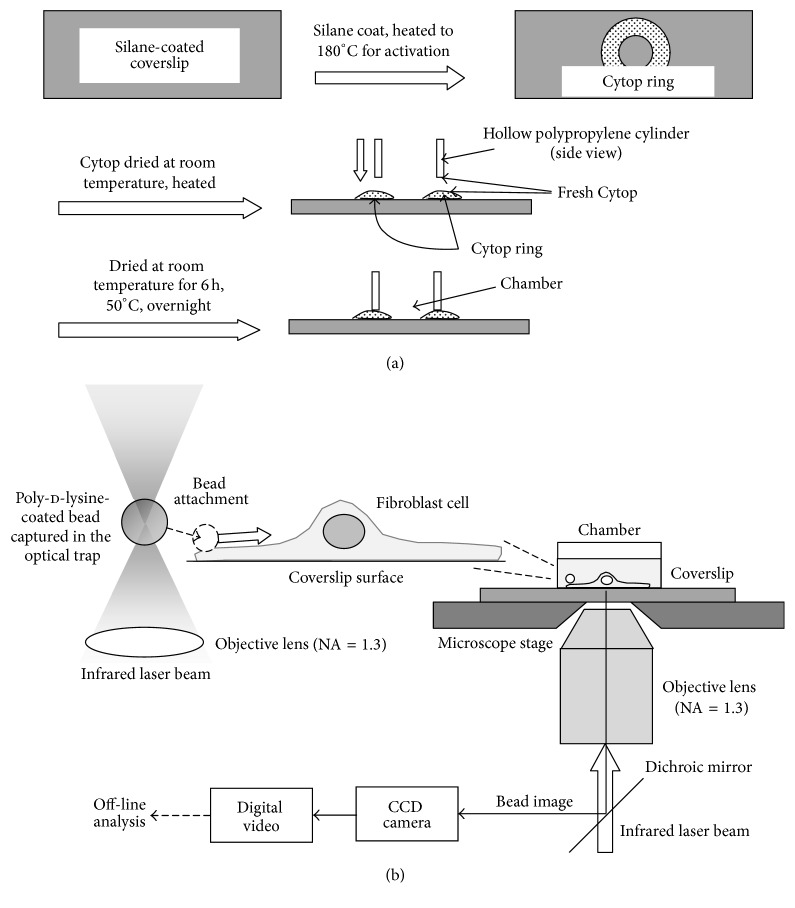
(a) Process of the construction of the Hirata chamber. The process after the coating of the coverslip with aminopropyltriethoxysilane is shown. See Section 2.2 for details. (b) The experimental set-up. An optical trap was generated in Zeiss inverted phase-contrast microscope by focusing an infrared laser beam (thick vertical arrow) with an objective lens (NA = 1.3). A poly-d-lysine-coated bead was captured in the trap and was attached to the edge of lamellipodium (the dashed arrow). As soon as the stable contact between the bead and the lamellipodium was achieved, the laser was turned off and the bead was allowed to move on the cell surface (bold horizontal arrow). The phase-contrast image of the bead was acquired with a CCD camera and was recorded on a digital video tape at 30 frames/sec. Video sequences were transferred to a hard disk for the off-line analysis (see Section 2.5). The figure is not drawn to scale.

Figure 1(a) summarizes the process of the construction of Hirata chamber. A no. 1 coverslip (24 mm × 60 mm) was coated with 3-aminopropyltriethoxysilane (2% in ethanol; Shin-Etsu Chemical Co., Ltd., Tokyo) at room temperature for 30 min. The silane-coating was activated by heating at 180°C for 30 min, and a fluorocarbon-based amorphous polymer (Cytop® CTX-809A, Asahi Technoglass, Shizuoka, Japan) was placed ring-wise at the center of the coverslip. After Cytop was dried at room temperature overnight, the coverslip was placed in an oven and was heated first at 50°C for 1.5 h and then at 180°C for 1 h. The mouth portion of a 15 mL polypropylene centrifuge tube (~10 mm height) was cut out, the bottom rim of which was applied with fresh Cytop; fresh Cytop was also applied to the top of the Cytop ring, and the tube was placed on the ring. Final drying process was carried out at room temperature for 6 h followed by an overnight incubation at 50°C.

### 2.3. Bead Coating

Polystyrene beads were coated with poly-d-lysine in 1 mg/mL poly-d-lysine aqueous solution at 4°C overnight. The coated beads were washed once in Milli-Q water to remove excess poly-d-lysine and were suspended in cell culture medium without FBS or phenol red but were supplemented with 10 mM HEPES (pH 7.2). Concanavalin A (50 *μ*g/mL~1000 *μ*g/mL) was covalently coupled to the polystyrene beads with 1-ethyl-3-(3-dimethylaminopropyl) carbodiimide. Amino groups of fibronectin III (7–10) were labeled with biotin-Sulfo-OSu and the modified fibronectin was bound to the avidin-bead through the biotin-avidin linkage.

### 2.4. Imaging of the Bead and the Cell

To measure the retrograde movement of the cell-bound bead, an inverted phase-contrast microscope (ICM401, Carl Zeiss, Tokyo, Japan), which was equipped with a 100x, NA = 1.3 objective lens, was used (Figure 1(b)). The optical trapping system implemented in this microscope [22] was utilized to attach the bead to the edge of lamellipodium, because it has been shown that the retrograde movement at the back of lamellipodium slowed down [23]. Phase-contrast image of the bead was acquired with a charge-coupled device (CCD) camera (C2400-7, Hamamatsu, Hamamatsu, Japan) and was recorded on a digital video tape. The recorded images were transferred to a hard disk for off-line analysis.

To visualize fluorescently stained cells, an inverted epi-fluorescence microscope (IX71, Olympus, Tokyo) equipped with a 100x, NA = 1.3 objective lens and a cooled CCD camera (C4742-96-12ERG, Hamamatsu, Hamamatsu, Japan) was used. The same camera was also used to acquire the phase-contrast images of the fluorescently stained cells (see below). The images were directly recorded on a hard disk for later analysis.

### 2.5. Measurement of the Retrograde Movement of the Bead and Analysis of the Bead-Trajectory with Mean-Square-Displacement Plot

In each experiment, the suspension of the poly-d-lysine-coated bead was added to the cell culture medium (1/1000~1/500 volume of the culture medium) in the Hirata chamber. The chamber was then transferred onto the stage of the microscope. To measure the retrograde movement, a bead was captured with the optical trap and the microscope stage was maneuvered to achieve the contact of the bead with the cell edge (within ~1 *μ*m from the edge of lamellipodium; Figure 1(b)). The use of the optical trap has an advantage, because one could choose the closest location to the lamellipodial edge as the attachment site. Usually within a few sec (at most 10 sec), the bead was firmly bound to the lamellipodium, and the laser was turned off to allow the bead to move on the surface of the lamellipodium. The whole sequence (bead capture, attachment, and transport) was recorded, as described above, and the trajectory of the bead was analyzed (see below).

We attempted to attach beads coated with concanavalin A or fibronectin III but could not achieve the firm binding; in both cases beads seemed to be only tethered to the cell and the turning off of the laser of the optical trap resulted in the movement of the bead away from the cell surface. Therefore, we used the poly-d-lysine beads.

Video image of the bead was used to determine the position of the bead every 33 ms with a program (“optical tweezers” written by Shoichi Ikeda) running on ImageJ (ver. 1.46) and was represented with (*x*(*t*_*j*_), *y*(*t*_*j*_)), where *t*_*j*_ = (*j* − 1)  *τ* and *j* varies from 1 (unity) to *N* (total number of the analyzed video frames) and *τ* = 33 ms, the minimum time interval, as described [22]. The *N* varied from 600 to 1400 depending on the experiment. The bead movements were not analyzed, if shrinkage of the lamellipodium or ruffling occurred during the observation (~25% of total experiments).

To analyze the bead motion, the mean-square-displacement (MSD) value was calculated from the bead position as follows:

(1) MSDkτ=1N−k·∑j=k+1Nxj−1·τ−xj−k−1·τ2+yj−1·τ−yj−k−1·τ2.

Here, *τ* = 33 ms and *N* and *k* represents integer ≥ 1. We assumed that the bead movement is a superposition of the two-dimensional lateral diffusion and a one-dimensional drift with *D* as the diffusion constant and *V* the time-independent velocity. On this assumption, MSD value is represented with the following equation:

(2) $$\begin{array}{c} MSD=4D\Delta t+V^{2}{\left(\;\Delta t\;\right)}^{2}, \end{array}$$

where Δ*t* ≡ *kτ* is the duration [24]. Thus, the bead movement is parametrized with two quantities,* D* and* V*.

Fluorescence speckle microscopy has been developed and applied to measure the turnover dynamics of actin filaments as well as the retrograde flow [25, 26]. With this technique hundreds of actin speckles can be analyzed with a spatiotemporal resolution of 270 nm and ~1 s, from which spatial variation of the rate of retrograde flow and that of actin turnover are derived. The bead analysis cannot provide the information of many moving objects, but the spatiotemporal resolution of the bead analysis is 10 nm and 33 ms, respectively, and is higher than that of the speckle microscopy. This feature is utilized to analyze the movement of the bead within Δ*t* < 0.1 s (Figure 3(d)).

### 2.6. Transient Expression of RFP-Actin Fusion Protein

To transiently express the RFP-actin in the cell, the plasmid was introduced into the cell with the transfection reagent, Lipofectamine 2000, according to the manufacturer's instruction.

### 2.7. Kymograph Analysis

Kymograph is also used to analyze the movements of cellular objects including the surface-bound bead, fluorescently-labeled actin filaments, or lamellipodia [22, 27]. We analyzed the dynamics of RFP-actin with this method: in lamellipodia containing RFP-actin, retrograde movements of the fluorescent spots were observed. From the video sequence the direction of the movement of the spots was identified and the kymograph was made along a straight line parallel to this direction. The unidirectional steady movement provided a straight line in the kymograph. With the calibration of the temporal and spatial axis, the slope of the straight line provided the velocity of the analyzed object. In an independent experiment, we also measured the velocity of the 1 *μ*m poly-d-lysine-coated bead attached to the cell expressing RFP-actin by the kymograph technique.

### 2.8. Inhibition of Actin Turnover and Myosin II Activity

To inhibit actin turnover, we used CytD, LatA, and Jasp. CytD binds to the fast-growing end of actin filament and inhibits the elongation of the filament [28]; LatA binds to actin monomer and sequesters it, thereby decreasing the rate of association of the monomer to the filament end [29]; Jasp inhibits the depolymerization of actin filaments [30]. For inhibition of myosin II, (−)-blebbistatin and Y-27632 were used. The mechanisms of the action of two inhibitors are different [31, 32]: the former inhibits the release of phosphate from the active site and the latter inhibits Rho-dependent kinase (ROCK), but both inhibit the actomyosin ATPase activity.

Quantification of the effect of inhibitors on *V*_fit_ was done as follows. After the addition of the inhibitor, cells were incubated for 30 min in the CO_2_ incubator. They were then subjected to the measurements and the analysis, as described in Section 2.5. The measurements of *V*_fit_ values of the untreated cells were carried out on the same day as the measurement with inhibitors, and the velocities obtained with inhibitors were always normalized to the average of the *V*_fit_ value of the untreated cells, because of the considerable variation of the *V*_fit_ values (see Section 3.1).

### 2.9. Cell Fixation and Fluorescent Staining of Actin Filaments

Fixation of the cell and fluorescent staining of actin filaments had been carried out according to the previously described method [25, 33, 34]: cells were fixed with 4% formaldehyde and then permeabilized with 0.1% Triton X-100. Actin filaments were visualized with 0.1 *μ*M rhodamine-phalloidin. Myosin II in the same cell was visualized by indirect immunofluorescence technique: mouse monoclonal antibody against myosin II light chain and Alexa488-labeled anti-mouse IgG were sequentially applied to the cell after fixation and permeabilization.

Profiles of the intensity of the fluorescence from actin filaments and that from myosin II were analyzed using ImageJ plugin “plot profile”. The numerical data were transferred to Microsoft Excel 2007 and were manipulated as follows. The intensity values of the fluorescence intensity obtained under each experimental condition were normalized to the maximum value in each profile. To determine the position where the profile exhibits a rapid rise, the derivative of the intensity profile was numerically determined and the position corresponding to the maximum value was assigned as the position of the rapid rise (see Section 3.3).

## 3. Results

### 3.1. Characterization of the Retrograde Movement of the Bead Attached to Lamellipodia

**Figure 2:**
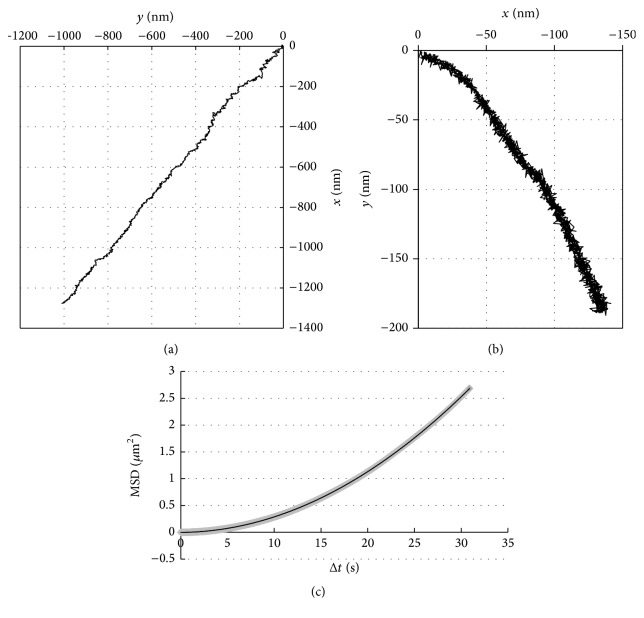
(a) The (*x*(*t*_*j*_), *y*(*t*_*j*_)) plots of a bead bound to lamellipodium of the untreated cell. The recording duration was 33 s. (b) The (*x*(*t*_*j*_), *y*(*t*_*j*_)) plots of a bead bound to the glass surface (fixed bead), representing the mechanical drift of the experimental system; the recording duration was 50 s. (c) The MSD plot of the bead moving on lamellipodium (see Section 2.5). The data were the same as that shown in (a); this curve was fit with a quadratic function of the time, Δ*t*: MSD = 0.002769Δ*t*^2^ + 0.001304Δ*t* − 0.001659, *R*^2^ = 0.999988.

An example of the* xy* trace of the bead obtained over 33 s is shown in Figure 2(a).  Figure 2(b) shows the* xy* trace of the bead bound to the glass surface, which was determined over 50 sec: this movement was due to the mechanical drift of the stage and the total distance traveled in this particular example was ~1/6 of that of the trajectory shown in Figure 2(a); the range of the movement of the fixed beads was always much smaller than the cell-bound beads (see below).  Figure 2(c) shows an example of the MSD plot. The curve-fitting provided *D* and *V* values as fitting parameters (represented with *D*_fit_ and *V*_fit_). In the example shown in Figure 2(c), *D*_fit_ = 272.3 nm^2^/s and *V*_fit_ = 52.7 nm/s, respectively.

**Figure 3:**
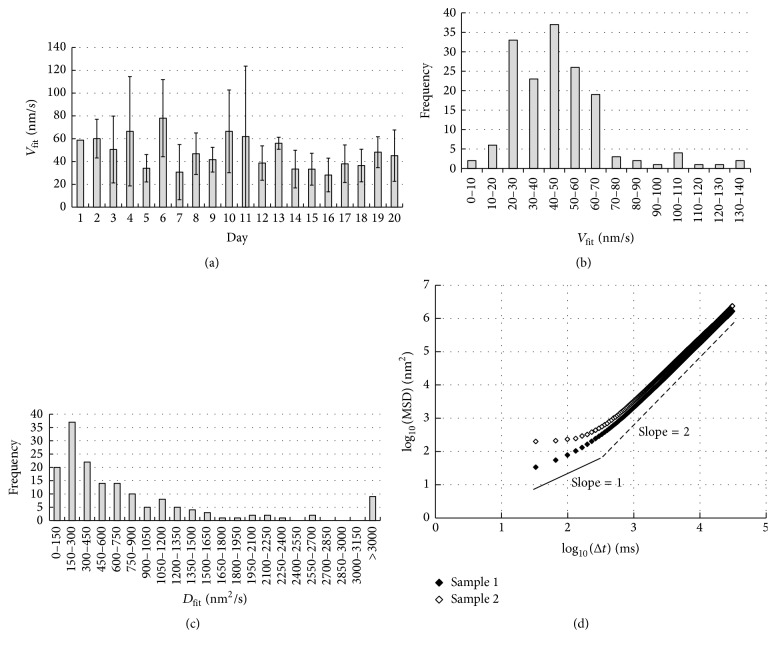
(a) Day-to-day variation of *V*_fit_ values of untreated cells. Total 160 measurements were carried out over 20 different days (on day 1 a single measurement was carried out). The average ± standard deviation of all the data was 46.6 ± 23.3 nm/s. (b) Distribution of the *V*_fit_ values shown in (a); majority of the *V*_fit_ values are <100 nm/s. (c) Distribution of *D*_fit_ values; the distribution is strongly skewed toward left and is quite broad. (d) log_10_(MSD) versus log_10_(Δ*t*) plot of an untreated cell (sample 1, closed symbols) and that of another untreated cell (sample 2, open symbols). The continuous straight line has a slope = 1 and the dashed straight line has a slope = 2, each of which represents a diffusion and a unidirectional movement with a constant velocity.

Figure 3(a) shows that the *V*_fit_ values measured on different days varied considerably.  Figure 3(b) shows the histogram of *V*_fit_ values obtained from the same set of the data as shown in Figure 3(a). A minor population of the data distributed >100 nm/s, but the major population distributed around 30–70 nm/s with the average value of 46.6 ± 23.3 nm/s (average ± standard deviation, 160 samples). The *V*_fit_ values were within the range of the velocities of the surface-bound beads, fluorescently-labeled actin, or intracellular vesicular bodies [7, 10, 23]. The average *V*_fit_ value of the beads fixed to the bottom of the chamber was 2.7 ± 1.8 nm/s (14 samples obtained in 5 independent experiments), which was much smaller than that of the control cells.

As shown in Figure 3(c), the distribution of the *D*_fit_ values skewed toward small values, implying that the diffusive movements very rarely occurred. Majority of the *D*_fit_ values were smaller than 10^3^ nm^2^/s and were almost two orders of magnitude smaller than the lateral diffusion coefficient of integrin *α*5*β*1 (5.3 ± 4.4) × 10^4^ nm^2^/s [34]. Previous studies have reported values of two-dimensional diffusion coefficient of a similar order of magnitude [10, 35]. Thus in the present case, the two-dimensional movement of the bead seemed to be restricted.

Figure 3(d) shows the plots of log_10_(MSD) versus log_10_(Δ*t*) for two samples (samples 1 and 2). The slopes of these plots for large Δ*t* (>1 s, or Log_10_Δ*t* > 3) were ~2, which is expected for the movement at a constant velocity, but the slope of the plot for small Δ*t* (<0.1 s; Log_10_Δ*t* < 2) was ~1 for sample 1 and ~0 for sample 2. Thus, in the latter sample, the bead seemed to execute subdiffusion, not Brownian movement. The subdiffusion is described with a relation between MSD and Δ*t*; MSD = *A*Δ*t*^*b*^, where *A* and* b* (between 0 and 1) are time-independent constants [36]. We speculate that the small *D*_fit_ values reflect the subdiffusional movements, which was not apparent from the MSD plot. At present, it is not possible to obtain more details of the movement for Δ*t* < 0.1 s, because 33 ms is the highest temporal resolution we can achieve. Hence, the reason for the variation is a theme of the future study.

**Figure 4:**
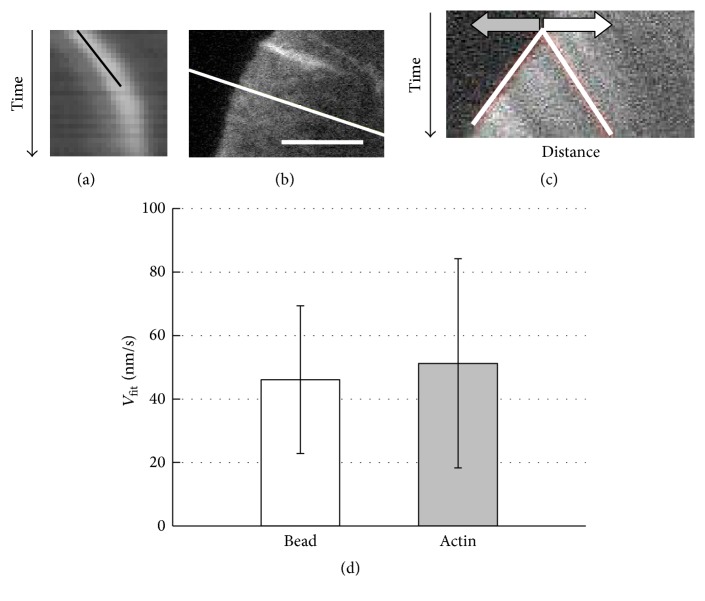
(a) A kymograph of 1 *μ*m bead moving on the surface of the cell expressing RFP-actin. The width of the vertical edge of the kymograph is 2.5 *μ*m and that of the horizontal edge is 27 s; the slope of the black line represents the velocity of the bead; for this particular example, the velocity was 87.5 nm/s. (b) Part of a lamellipodium of the cell expressing RFP-actin; kymograph of RFP-actin was generated along the white line. Horizontal bar, 5 *μ*m. (c) The kymograph; gray arrow indicates the forward motion of the lamellipodial edge, and white arrow indicates the retrograde movement of the fluorescence pattern of RFP-actin. The width of the vertical edge is 60 sec and that of the horizontal edge is 16 *μ*m. In this case, protrusion of lamellipodium and retrograde flow of actin occurred at the same time. The slopes of two white lines represent the velocity of the bead and the cell edge. (d) The average of the velocity of the bead bound to the lamellipodium of the cell expressing RFP-actin (62 measurements) and that of RFP-actin (87 measurements), both estimated by kymograph method. Error bar, standard deviation.

We next compared the velocity of the bead, which was attached to the cell expressing RFP-actin, with the velocity of the RFP-actin in lamellipodium. Two measurements were carried out in different cells, because in our hand it was not possible to bring two objects simultaneously into the focus. Similar comparison has been made in several previous studies [7–9].  Figure 4(a) represents an example of the kymograph generated from a sequence of phase-contrast image of the bead. The black line was drawn to trace the kymograph. This kymograph curved toward the end of the record, because the bead gradually went out of focus; the curved part was not used for the analysis.  Figure 4(b) shows a lamellipodium of the cell expressing RFP-actin. The white line indicates the direction along which the kymograph was generated.  Figure 4(c) represents an example of the kymograph, in which two movements are apparent: one is the retrograde movement of the actin spot (indicated with the horizontal white arrow) and the other is the protrusive movement of the cell edge (horizontal gray arrow). The velocity of the retrograde movement of RFP-actin was calculated from the straight line drawn below the white arrow.  Figure 4(d) shows the comparison of the velocity of the bead and that of the RFP-actin: the velocity of the bead was 46.1 ± 23.3 nm/s (average ± standard deviation; 62 samples), and that of the RFP-actin was 51.2 ± 32.9 nm/s (87 samples). The difference between the two values was not statistically significant (*p* > 0.05, two-tailed* t*-test). Thus, we conclude that the velocity of the retrograde movement of actin filaments was the same as that of the surface-bound bead.

### 3.2. Effect of the Inhibition of Actin Turnover/Myosin II Activity on the Retrograde Movement

**Figure 5:**
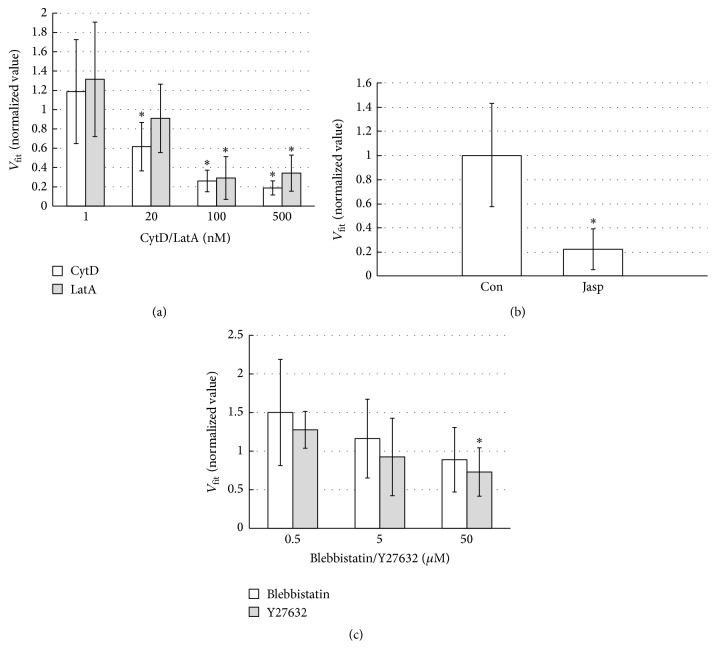
(a) The effect of CytD/LatA on the average *V*_fit_ values; error bar, standard deviation. (b) Comparison of the average *V*_fit_ values of the control cells and the cells treated with 0.5 *μ*M Jasp. (c) The effect of (−)-blebbistatin/Y-27632 on *V*_fit_ values; error bar, standard deviation. In (a) and (c), *V*_fit_ values were normalized to the control values, which were determined on the same day when each inhibitor experiment was carried out. Asterisks in (a) to (c) indicate the statistically significant change (*p* < 0.05) of the average *V*_fit_ values from that of the untreated cells.

We investigated how the inhibition of actin dynamics affected the retrograde movement of the bead.  Figure 5(a) shows *V*_fit_ of the bead bound to the surface of the cell treated with CytD (open bars) or LatA (gray bars). The *V*_fit_ values were normalized to the *V*_fit_ values of the untreated cells measured on the same day as the day of the inhibitor experiment to compensate for the day-to-day variability of the *V*_fit_ values (Figure 3(a)). In the presence of CytD ≥20 nM, the *V*_fit_ values were significantly lower than the control values (*p* < 0.05, indicated with asterisks). The *V*_fit_ values in the presence of LatA ≥100 nM were significantly lower than the control values (*p* < 0.05, indicated with asterisks). As shown in Figure 5(b), 0.5 *μ*M Jasp strongly lowered the velocity of the retrograde bead movement: the average *V*_fit_ value in the presence of Jasp was 15 ± 13 nm/s and was significantly lower than the control value (68 ± 29 nm/s; *p* < 0.05, 6 samples). These results altogether indicate that the actin turnover plays an important role in the retrograde movement of the surface-bound bead.

We next examined the effect of the inhibitors of myosin II activity ((−)-blebbistatin and Y-27632) on the *V*_fit_ values (Figure 5(c)). The average *V*_fit_ value in the presence of 50 *μ*M (−)-blebbistatin was lower than the control value by ~11%, but the difference was not statistically significant (*p* > 0.05). The *V*_fit_ obtained at 50 *μ*M Y-27632 was significantly lower than that of the control value by ~27% (*p* < 0.05). Thus, the inhibition of myosin II activity only slightly slowed the retrograde movement of the surface-bound beads.

### 3.3. Effect of Actin or Myosin II Inhibitors on the Intracellular Distribution of Actin Filaments and Myosin II

**Figure 6:**
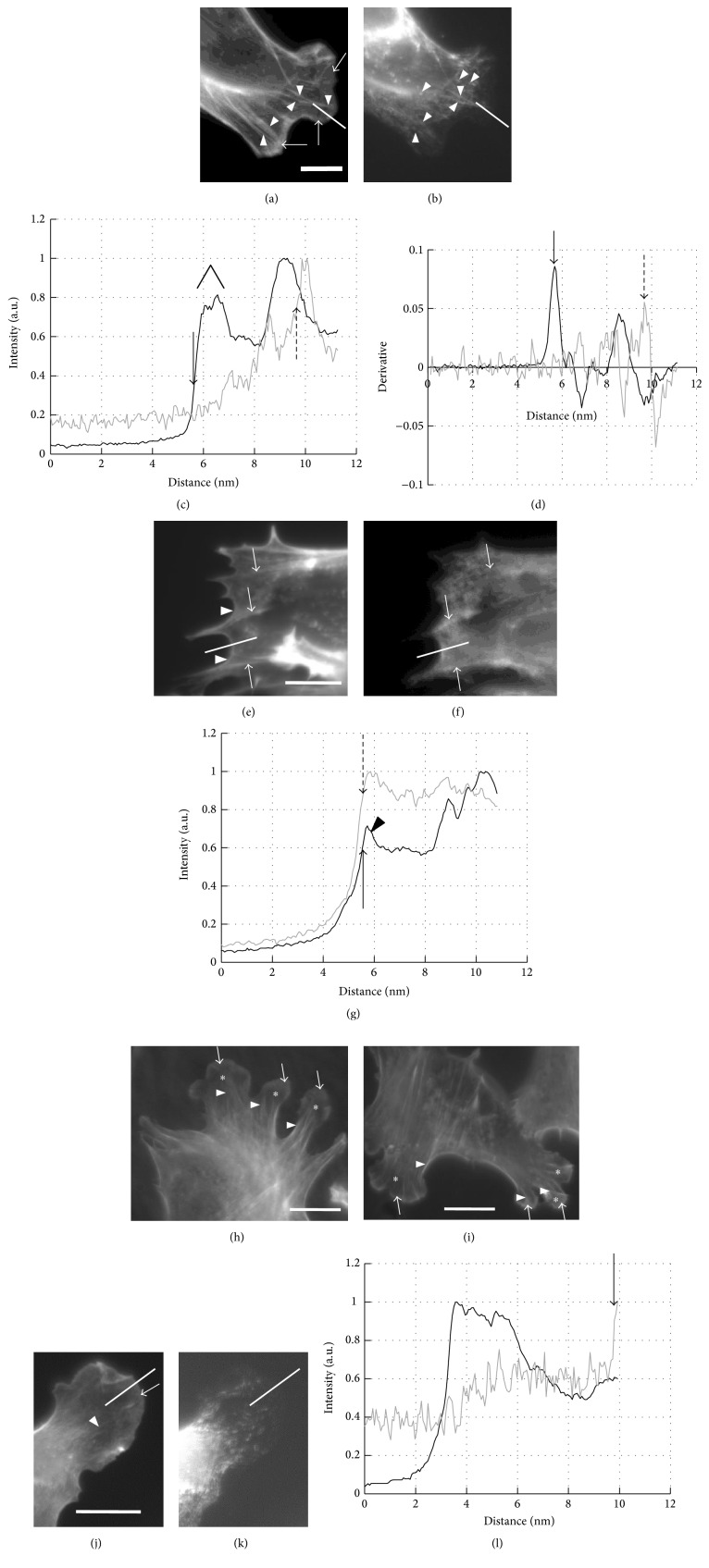
Effect of the inhibitors of actin turnover or those of myosin II activity on the distribution of actin filaments and myosin II. Under each condition, distributions of actin and myosin II are compared in the same cell. (a) Actin filaments in the control cell; arrows, vague staining of actin filament network in the lamellipodium; arrowheads, actin bundles. (b) Myosin II in the same cell; arrowheads indicate the spot-like fluorescence attributed to myosin II mini-filaments, some of which reside along the actin bundles. Unlike actin, distribution of myosin II along the cell periphery was hardly observed. (c) Comparison of fluorescence intensity of actin filaments and that of myosin II measured along the white lines shown in (a) and (b). The fluorescence intensity was normalized as described in Section 2.9. The arrow and the dashed arrow indicate the start of the rapid rise of the profile of actin and that of myosin II, respectively. The symbol (∧) indicates the plateau corresponding to the vague actin staining. (d) Derivative of the intensity profiles shown in (a) and (b); the arrow and the dashed arrow indicate the positions of local maxima of the derivative of the profiles of actin and that of myosin II, each of which corresponds to the rapid rise of each profile. (e) Actin filaments in the cell treated with 100 nM CytD; arrows indicate the distribution of actin bundles (i.e., stress fibers); arrowheads indicate the cell edge where rim-like distribution of actin filaments is seen. (f) The same cell stained for myosin II; there is some colocalization of myosin II with actin bundles (arrows). (g) Line profiles of fluorescence intensity for actin (black line) and myosin II (gray line) measured along the white lines shown in (d) and (e). The arrow and the dashed arrow indicate the start of the rapid rise of the profiles of actin and myosin II, respectively. The arrowhead indicates a peak of the fluorescence profile corresponding to the actin rim. ((h) and (i)) Distribution of actin filaments in the cell treated with 5 *μ*M (−)-blebbistatin or with 5 *μ*M Y-27632, respectively. Asterisks indicate lamellipodia; arrows in both panels indicate the vague staining of actin in lamellipodia; arrowheads in both panels indicate actin stress fibers. ((j) and (k)) Distributions of actin filaments and myosin II in the cell treated with 50 *μ*M (−)-blebbistatin. Arrows indicate the vague staining of actin and arrowheads indicate actin bundles. (l) Line profiles of the intensity of actin filaments (black line) and that of myosin II (gray line) measured along the white lines shown in (i) and (j), respectively; the background (outside the cell) of the image field for myosin (k) was higher than that of actin probably because of the nonspecific binding of the secondary antibody to the glass surface. The wide plateau corresponding to the vague actin fluorescence is evident between ~3.5 *μ*m and ~5.5 *μ*m from the start of the profile plot. White thick bar in (j) indicates 10 *μ*m.

Figure 6(a) shows the fluorescence micrograph of an untreated cell stained for actin, and Figure 6(b) shows the same cell but immuno-stained for myosin II. Comparison of the two panels indicates that the pattern of the distribution of actin filaments and myosin II was different especially in lamellipodium. Figure 6(a) shows that actin in the lamellipodium exhibits somewhat graded, vague staining (arrows). Actin bundles (arrowheads) are penetrating through the vague staining. The vague staining of actin in lamellipodium of fibroblasts and B16-F1 mouse melanoma cells has been attributed to the dense meshwork of actin filaments, which has been supported by the electron microscopic observation [3, 12]. Lamellipodia of the cells transiently expressing RFP-actin exhibited similar pattern of fluorescence in lamellipodium (not shown). As shown in Figure 6(b), myosin II appeared as spots. Some spots were on the actin bundles and the rest of the spots were at the back of lamellipodium (arrowheads in Figure 6(b)). The latter observation is consistent with the previous observation that myosin II was distributed mostly in lamella as bipolar mini-filaments [18]. Colocalization of myosin II with the actin bundle demonstrates that the bundle is a stress fiber [27].

The different distribution of actin filaments and myosin II in lamellipodium is clearly demonstrated in the different position of the rapid rise of line profiles (Figure 6(c); black line for actin, gray line for myosin II). The position of rapid rise of the actin intensity, as determined by the method described in Section 2.9, is shown with the arrow in Figure 6(d) and that of myosin II intensity is shown with the dashed arrow. The black arrow indicates the position of the most rapid rise of actin intensity, and the dashed arrow indicates that of myosin II intensity. The different positions of these arrows demonstrate the difference in the distribution of actin and myosin filament. Similar differential distribution has been previously reported [37, 38]. Immediately behind the position of the rapid rise of actin profile, a plateau (indicated with ∧) appears: this corresponds to the vague staining of actin near the cell edge.

Figure 6(e) shows that 100 nM CytD drastically altered the distribution of actin filaments. The vague staining along the edge of lamellipodium disappeared; instead, the edge was rimmed by actin filaments (arrowheads). Number of stress fibers seemed to be decreased, and the remaining actin bundles (arrows) made spikes. The region where myosin II existed overlapped the region where actin was populated (Figure 6(f); arrows indicate the same actin bundles as those in Figure 6(e)). As demonstrated in the line-profile plot (Figure 6(g)), the rise of the actin and myosin profiles started at almost the same positions (arrow for actin and dashed arrow for myosin II, determined in the same manner as described above; the arrowhead indicates the position of the actin rim). Similar distributions of actin and myosin II were observed in the cell treated with LatA (not shown).

Majority of the cells treated with 5 *μ*M (−)-blebbistatin or 5 *μ*M Y-27632 (Figures 6(h) and 6(i)) possessed lamellipodia (asterisks). Individual lamellipodia were narrower and longer than those of the untreated cells, and the number of lamellipodia in individual cells increased. Elongated lamellipodia of* Aplysia* bag-cell neuronal growth cone treated with 70 *μ*M (−)-blebbistatin had been demonstrated [23]. The vague staining of actin along the cell edge was observed (white arrows in Figures 6(h) and 6(i)), as in the untreated cell. The number of stress fibers seemed to be decreased and many of them were thinner than those in the untreated cells (Figures 6(h) and 6(i), arrowheads). The stress fibers behind the lamellipodia (arrowheads) are longer than those in the untreated cell; elongation of stress fibers has been observed in the* Aplysia* growth cone treated with (−)-blebbistatin, which has been interpreted as the decrease in the severing activity of myosin II (see Section 4).

Figures 6(j) and 6(k) show the distribution of actin filaments and myosin II in the cell treated with 50 *μ*M (−)-blebbistatin. The peripheral vague staining still existed, but the stress fibers penetrating the lamellipodium almost disappeared (Figure 6(j); arrowhead indicates very faint line) as compared with the cell treated with 5 *μ*M (−)-blebbistatin. On the other hand, the distribution of myosin II was quite similar to that in the untreated cell and did not overlap that of actin. Thus, the line profiles of actin and myosin (see Figure 6(l)) measured along the white line shown in Figures 6(j) and 6(k) demonstrate that the distribution of actin started rising at the cell periphery, whereas that of myosin II rose slowly and exhibited a sharp rise only at the back of lamellipodium (arrow in Figure 6(l)). The plateau in the profile plot is evident between ~3.5 *μ*m and ~5.5 *μ*m from the start of the profile. We note that the (−)-blebbistatin at 50 *μ*M, which is ten times higher concentration than that used for the cell shown in Figure 6(h), provided similar narrow and elongated morphology of lamellipodia; we consider that this argues against the possibility of the photoinactivation of (−)-blebbistatin [39] or of insufficient concentration of the drug. The possibility is also remote that the concentration of Y-27632 was not sufficiently high, because we previously showed that 10 *μ*M Y-27632 strongly inhibited the contraction of stress fibers in Swiss 3T3 fibroblast [27].

## 4. Discussion

It has been postulated that the retrograde movements occur as a result of either the reaction of the pushing of the growing actin filament network against the edge of lamellipodium, myosin II-based contraction of actin filament network in a lamellum behind lamellipodium, or both. To investigate the relative contribution of actin polymerization and myosin II activity, we measured the velocity of the retrograde movement of the poly-d-lysine-coated bead attached to the tip of lamellipodium and compared the velocities in the presence and absence of the inhibitors of actin turnover or myosin II activity.

Inhibition of actin turnover with CytD, LatA, or Jasp greatly slowed down the retrograde movement of the bead. On the other hand, inhibition of myosin II ATPase activity slightly lowered the velocity. Thus, in Swiss 3T3 fibroblasts actin turnover played a major role in the retrograde movement, whereas myosin II activity played secondary or some other roles.

As a result of the inhibition of the actin polymerization by CytD or LatA, the vague staining of actin filament network at the edge of lamellipodium disappeared. This was because the actin polymerization was inhibited by either the barbed-end capping activity of CytD [28] or the actin-sequestering action of LatA [29], whereas the depolymerization of actin at the back of lamellipodium was not affected by these inhibitors [14]. The significant decrease of the *V*_fit_ value in the presence of these inhibitors can be explained as a result of the loss of actin network structure.

In the CytD- or LatA-treated cells rim of actin appeared along the cell edge. A previous study [3] has proposed a mechanism of the generation of similar structure: during a pause of the protrusion of lamellipodium, short filaments disappear as a result of the depolymerization activity. Longer filaments that have survived the depolymerization form bundles with myosin II. As some of the actin filaments in this bundle are connected to the actomyosin network in lamella, the contraction of the actomyosin will pull the cell edge. With the attachment of both ends of the bundle to the cell adhesion sites, an inward curvature of the bundle will be generated. We presume that the rim observed in the present study was formed in an analogous process, because actin polymerization was halted by actin inhibitors, but both the cell-specific depolymerization activity and myosin II activity were not changed.

After the treatment of the cell with CytD or LatA, stress fibers seemed to be diminished but did not totally disappear. It has been suggested that stress fibers are formed by a connection between actin filaments that elongated by polymerization from the cell adhesion site and those formed at the cell periphery and joined together by myosin II filaments [40] or those formed by bundling of actin filaments severed in lamellipodium (a cytoplasmic pool of actin filaments) [41]. Thus, CytD/LatA, by inhibiting the formation of new filament, would have depleted the filament pool, and, hence, the formation of stress fibers by the coalition process was inhibited, leading to the diminution. On the other hand, it has been shown that mutual interaction between adjacent actin filaments in a stress fiber is stabilized by multiple proteins such as *α*-actinin, Arp2/3, or bipolar myosin filament [42]. Probably because of this, the on-and-off rates of actin monomer to and from a stress fiber are low [40]. As a result, some stress fibers remained after CytD/LatA treatment. A study with MTF24 fibroblast has shown that stress fibers remained after the treatment of the cell with 100 nM CytD for 2 h [43].

The inhibition of myosin II activity by (−)-blebbistatin or Y-27632 resulted in only a small decrease of *V*_fit_ values even at the highest concentration (50 *μ*M) of these drugs. The actin network structure along the cell edge still existed, which is represented with the wide plateau in the line profile of actin staining. We assume that the remaining actin network was driving the retrograde movements. On the other hand, stress fibers in the cells treated with these drugs were progressively lost (Figure 6(h) for 5 *μ*M (−)-blebbistatin and Figure 6(j) for 50 *μ*M (−)-blebbistatin). This was probably due to the decrease in contractility of the stress fiber [44, 45]. The development of longer lamellipodia in the cells treated with (−)-blebbistatin/Y-27632 was a combined result of the decreased contractility and the elongation of actin by polymerization that was not affected by the myosin II inhibitors [14, 23]. The reason for the narrowing of lamellipodia and the increase in their number is not clear.

Our experimental results collectively suggest that the actin polymerization is the major source of the driving force of the retrograde flow, while the contractile activity of myosin II may play some other roles. It has been suggested that filament formation by actin polymerization at the front of the actin network and the depolymerization at the back, which follows the severing of filaments, is balanced [46]. These two processes are mediated by the retrograde flow of actin network with severing and the forward transport of actin monomer [47]. It has been suggested that the contractile force generated by myosin II filaments promotes the severing and/or depolymerization of actin filaments [23]. If this occurs in our system, the inhibition of the activity of myosin II will decrease the amount of the actin returned to the leading edge. As a result, the polymerization rate and hence the velocity of retrograde flow will be decreased. We propose that the slight decrease in the *V*_fit_ in the presence of 50 *μ*M (−)-blebbistatin or Y-27632 was due to the impairment of the severing function.

Previous studies have demonstrated that the retrograde flow depends on the activity of myosin [9, 21, 48]. Involvement of myosin 1c [9] or myosin IIA [48] in the retrograde flow has been demonstrated. In these studies the pulling action of myosin is considered to be the most important. Our study has suggested that myosin II participates in the process of retrograde flow in a way different from what has been demonstrated in the above studies. However, it is also possible that some intricate mechanism exists that compensates the loss of myosin II activity in the retrograde flow. Further study is necessary to fully understand the relative importance of the two mechanisms in the retrograde flow.
